# Polymorphic pseudogenes in the human genome - a comprehensive assessment

**DOI:** 10.1007/s00439-024-02715-9

**Published:** 2024-11-02

**Authors:** Mónica Lopes-Marques, M. João Peixoto, David N. Cooper, M. João Prata, Luísa Azevedo, L. Filipe C. Castro

**Affiliations:** 1 CIIMAR-Interdisciplinary Centre of Marine and Environmental Research, University of Porto, Porto, Portugal; 2 Institute of Medical Genetics, School of Medicine, Cardiff University, Cardiff, UK; 3 Instituto de Investigação e Inovação em Saúde, University of Porto, Porto, Portugal; 4 FCUP- Faculty of Sciences, Biology Department, University of Porto, Porto, Portugal; 5 UMIB-Unit for Multidisciplinary Research in Biomedicine, ICBAS - School of Medicine and Biomedical Sciences, University of Porto, Porto, Portugal; 6 ITR - Laboratory for Integrative and Translational Research in Population Health, Porto, Portugal

**Keywords:** Polymorphic pseudogene, Gene loss, Loss of function, Population analysis, Gene essentiality

## Abstract

Background: Over the past decade, variations of the coding portion of the human genome have become increasingly evident. In this study, we focus on polymorphic pseudogenes, a unique and relatively unexplored type of pseudogene whose inactivating mutations have not yet been fixed in the human genome at the global population level. Thus, polymorphic pseudogenes are characterized by the presence in the population of both coding alleles and non-coding alleles originating from Loss-of-Function (LoF) mutations. These alleles can be found both in heterozygosity and in homozygosity in different human populations and thus represent pseudogenes that have not yet been fixed in the population. Results: A methodical cross-population analysis of 232 polymorphic pseudogenes, including 35 new examples, reveals that human olfactory signalling, drug metabolism and immunity are among the systems most impacted by the variable presence of LoF variants at high frequencies. Within this dataset, a total of 179 genes presented polymorphic LoF variants in all analysed populations. Transcriptome and proteome analysis confirmed that although these genes may harbour LoF alleles, when the coding allele is present, the gene remains active and can play a functional role in various metabolic pathways, including drug/xenobiotic metabolism and immunity. The observation that many polymorphic pseudogenes are members of multigene families argues that genetic redundancy may play a key role in compensating for the inactivation of one paralogue. Conclusions: The distribution, expression and integration of cellular/biological networks in relation to human polymorphic pseudogenes, provide novel insights into the architecture of the human genome and the dynamics of gene gain and loss with likely functional impact.

**Supplementary Information:**

The online version contains supplementary material available at 10.1007/s00439-024-02715-9.

## Background

The first draft sequence of the human genome was published more than two decades ago (Lander et al. 2001). Recently, the publication of the polished Telomere-to-Telomere sequence completed key aspects of the project, including all centromeric satellite arrays (Nurk et al. 2022). Even so, many features of our genome remain enigmatic. For example, the precise number of functional protein-coding genes in the human genome remains elusive. This number has been subject to considerable debate, with currently accepted estimates ranging from 19,000 to approximately 22,000 genes (Amaral et al. 2023). Contributing to this uncertainty is interindividual genetic variation, with the implication that the exact number of protein coding genes varies between individuals (Abascal et al. 2018; Rausell et al. 2020; Auton et al. 2015). This can be due to structural variants (gross insertions, gross deletions, inversions and copy number variants) in genomic regions encompassing protein-coding genes (Auton et al. 2015) or to the existence of polymorphic pseudogenes. Previous studies have offered several distinct yet analogous definitions of polymorphic pseudogenes. Thus, the GENCODE project defined them as protein coding genes that have been pseudogenized by the presence of polymorphic truncations (Pei et al. 2012) whereas Zhang et al. described them as genes with polymorphic disruptive sites with both functional and non-functional alleles segregating in the population (Zhang et al. 2010). More recently, Abascal et al. (2018) defined polymorphic pseudogenes as pseudogenes that are intact (i.e. not inactivated) in some individuals (Abascal et al. 2018) whilst Rausell et al. (2020) defined them as genes characterized by frequent homozygosity for loss-of-function (LoF) variants (Rausell et al. 2020). In terms of population genetics, these definitions all imply that both the LoF allele and coding allele are segregating in the general population. Since individuals homozygous for the LoF allele do not usually manifest a clinical phenotype, polymorphic pseudogenes have generally been assumed to represent non-essential (or dispensable) genes, as opposed to those genes deemed to be essential, in which the loss of function compromises viability or results in severe fitness effects due to the gene’s role in key physiological processes (Rausell et al. 2020; Bartha et al. 2018). We should however appreciate that the precise distinction between essential and non-essential genes is rather vague and subject to external factors, as selection may act differently on the same LoF mutation depending upon the environmental context. Up to this point there has been a general consensus (Rausell et al. 2020; Bartha et al. 2018) that polymorphic pseudogenes likely fall into the non-essential category and that the LoF mutations in these genes are not expected to impact individual fitness, even in cases of homozygosity.

In this work, we took into account previous descriptions of polymorphic pseudogenes (Abascal et al. 2018; Rausell et al. 2020; Pei et al. 2012; Zhang et al. 2010) and include a minimum frequency requirement for each LoF allele so that it is represented in the general population. Thus, here we define polymorphic pseudogenes as genes which may (or may not, depending upon individual variation) carry a LoF allele that occurs with a frequency higher than 1% (in global or specific sub-populations) and in homozygosity with no overt pathogenic consequences.

A classic example of a polymorphic pseudogene is the blood group *ABO* gene. Indeed, whilst two of the most frequent alleles of the *ABO* gene encode enzymes with glycosyltransferase activity that underlie the blood group phenotypes A, B and AB, a third allele is a pseudogenized sequence due to the introduction of a LoF frameshift mutation leading to a premature stop codon (rs8176719; c.261del, pThr88Profs*31) that, in homozygosity, results in the O blood group (Yamamoto et al. 1990). The *RHD* gene, which determines the rhesus blood type Rh-positive or Rh-negative phenotypes, also constitutes a polymorphic pseudogene since it contains multiple Open Reading Frame (ORF)-disrupting mutations including full and partial gene deletions, some of which originated during the evolution of the hominids (Flegel 2007; Wagner and Flegel 2000). Determination of the blood type under the control of these two polymorphic pseudogenes is essential to guarantee successful blood transfusions and, in the case of the Rh antigen, to assess histocompatibility during pregnancy.

Various other polymorphic pseudogenes have been previously identified in humans, for instance, in the *CCR5* (Samson et al. 1996) *GBA3* (Lopes-Marques et al. 2020), *CASP12* (Fischer et al. 2002; Yeretssian et al. 2009), *NAT8B* (Veiga-da-Cunha et al. 2010) and *SERPINA2* (Marques et al. 2013) loci. Although explanatory models for the high frequency of LoF variants for some polymorphic pseudogenes have suggested a relationship between gene loss and dietary preferences (e.g. *GBA3*) or resistance to pathogens (e.g. *CASP12*), in most cases the modulating factor has yet to be identified (Lopes-Marques et al. 2020; Fischer et al. 2002; Yeretssian et al. 2009).

Among the currently annotated human polymorphic pseudogenes, those belonging to the gene superfamily that encodes olfactory receptors (OR) stand out numerically (Abascal et al. 2018; Rausell et al. 2020). The OR superfamily is the largest multigene family in vertebrates, representing a rapidly evolving family of genes that has experienced many expansions and contractions during mammalian evolution, probably in response to species-specific sensory requirements (Bear Daniel et al. 2016). In humans, the OR gene family has been estimated to comprise 802 distinct genes of which approximately 415 (51.7%) are fixed pseudogenes (Niimura and Nei 2007). This pronounced gene inactivation has been proposed to have occurred concurrently with the enhancement of the visual system and diminution of the olfactory sense during primate evolution (Niimura and Nei 2007; Lucas et al. 2003).

Although the lack of an observable associated phenotype has often hampered the identification of human polymorphic pseudogenes and prevented us from understanding their biological meaning, previous studies have begun to explore polymorphic pseudogenes, thereby generating valuable information on the topic. In most cases, these studies have been focused on a single gene e.g. *GBA3* (Lopes-Marques et al. 2020), *CCR5* (Samson et al. 1996) and *GPR33* (Römpler et al. 2005), although other studies, namely those of Abascal et al. (Abascal et al. 2018), and Rausell et al. (Rausell et al. 2020), succeeded in identifying larger sets of polymorphic pseudogenes. In this work, we present an exhaustively curated dataset of human polymorphic pseudogenes. In addition to previously identified polymorphic pseudogenes, we include 35 newly discovered polymorphic pseudogenes identified in the present study. Interrogation of this unified dataset then allowed us to obtain novel insights into the main tissues, metabolic pathways and physiological features impacted by the loss of functional alleles in these genes from the human genome.

## Results

### Collation and validation of human polymorphic pseudogenes

**Fig. 1:**
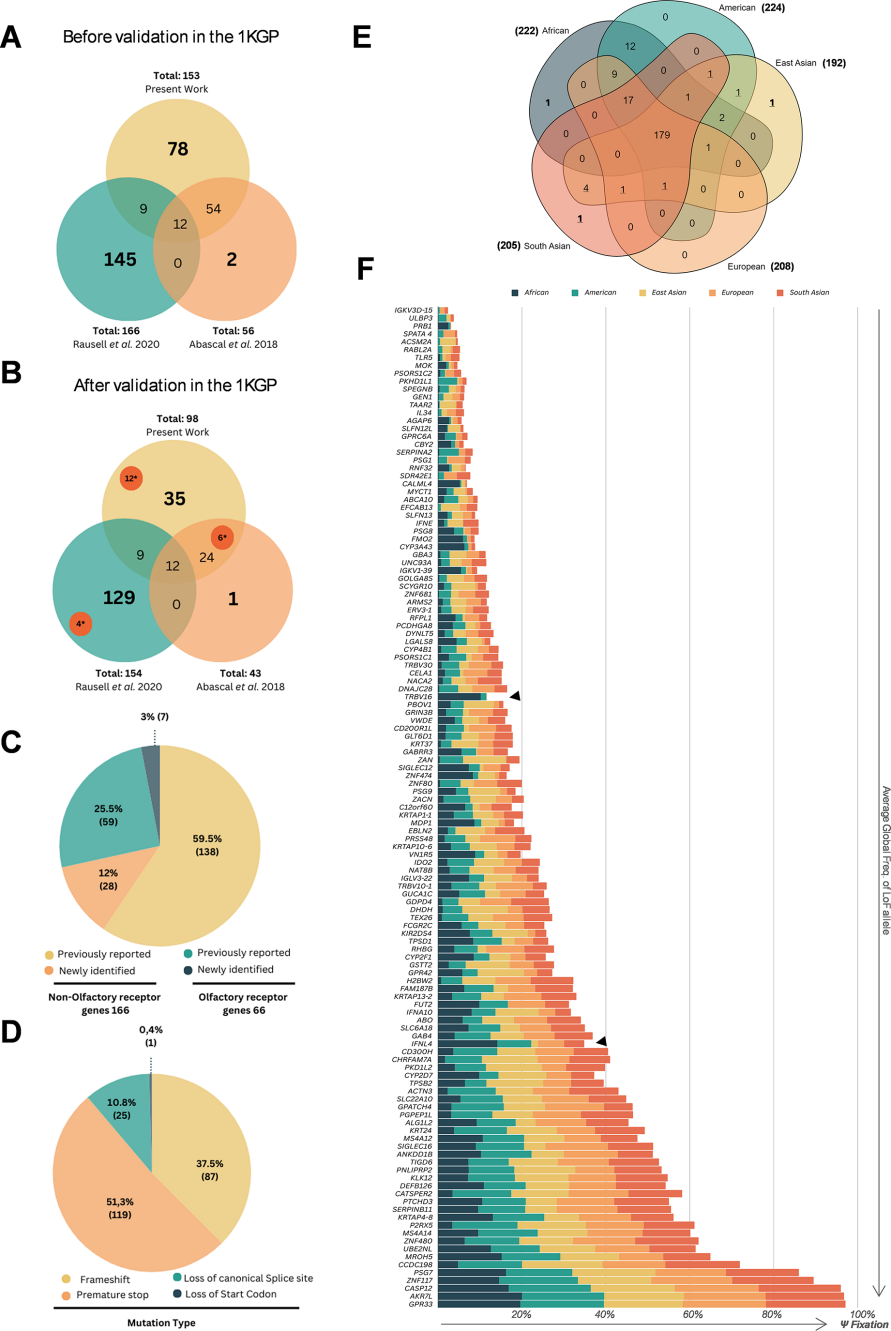
**A** - Venn diagram showing the number of polymorphic pseudogenes identified in each study, **B** - Venn diagram displaying the number of polymorphic pseudogenes with a LoF allele and at least one homozygous individual in the 1KGP populations. *Highlights genes identified previously in independent reports (see Additional Table 1). **C** - Number of OR and non-OR polymorphic pseudogenes previously identified and newly identified in this work. **D** - Main types of ORF-disrupting mutations identified. **E** - Venn diagram depicting the distribution of polymorphic pseudogenes among the 1KGP populations, numbers in bold correspond to genes in which the LoF allele only appeared in one specific population, numbers underlined correspond to gene in which the LoF allele was not present in the African population. **F -** Stacked bar graph of non-OR polymorphic pseudogenes and frequencies of LoF allele in the 1KGP genomes subpopulations, values on the X axes refer to the averaged frequencies of the LoF alleles across 5 populations

From our initial searches in public databases (NCBI RefSeq collection and GeneCards^®^), a total of 153 genes were identified and collated. We then merged our findings with those of Abascal et al. (Abascal et al. 2018) and Rausell et al. (Rausell et al. 2020) yielding a single non-redundant list of 300 genes (Additional Table 1). When merging the findings from these three sources, we identified an overlap of 12 genes between them (all 12 of these genes encoded olfactory receptors), with 9 and 54 genes having been previously identified by Rausell et al. (Rausell et al. 2020) and Abascal et al. (Abascal et al. 2018) respectively (Fig. 1A, Additional Table 1). It is important to note that the criteria used to identify polymorphic pseudogenes differed slightly between studies. Whilst Abascal et al. identified these genes by searching for discrepancies in the classification of coding status between three databases and did not evaluate allele frequencies (Abascal et al. 2018), in the present work and in the work of Rausell et al. (2020), a relatively similar strategy was used which involved the identification of LoF alleles and the evaluation of their corresponding frequencies. The work of Rausell et al. (Rausell et al. 2020). differed from the present work with regard to two key points, namely the databases searched (GnomAD vs. 1000 Genomes Project Phase 3 (1KGP), respectively) and the minimum frequency of homozygotes required (1% homozygotes vs. at least one homozygote, respectively). Next, to standardise the criteria for the identification of polymorphic pseudogenes, we manually investigated and validated each candidate polymorphic pseudogene so as to identify the most frequent LoF mutation present in the 1KGP and then collated the allele and genotype frequencies for each LoF allele. This strategy led to the exclusion of 68 genes from the initial list due to: (i) their being annotated as pseudogenes (i.e. distribution of LoF allele/alleles has attained 100% frequency in the global population) in the analysed human genome assembly GRCh38.p14 (24 genes) or encoding an lncRNA (3 genes), (ii) the absence of an LoF mutation in the 1KGP (16 genes) and (iii) the lack of at least one individual homozygous for LoF mutation in the 1KGP (25 genes) (Fig. 1B, Additional Fig. 1).

The remaining 232 genes were considered *bona fide* polymorphic pseudogenes with both LoF and coding alleles segregating in one or more human populations. These 232 polymorphic pseudogenes were divided into two groups, the first comprising all olfactory receptor (OR) genes and the second containing the remaining (non-OR) genes. The first group comprised 66 OR genes (Glusman et al. 2001; Malnic et al. 2004), including seven new polymorphic pseudogenes: *OR1P1*,* OR3A1*,* OR4C45*,* OR4K3*,* OR4L1*,* OR52P1* and *OR8G1* (Fig. 1C). The non-OR group comprised 166 polymorphic pseudogenes, 28 of which are reported for the first time as polymorphic pseudogenes in this study (Fig. 1B; Table 1). Overall, the most frequent type of LoF mutation was protein truncation due to the introduction of premature stop codons. This was followed by frameshift mutations, then mutations that abolished canonical splice sites, and finally one example of the loss of the start codon (Fig. 1D).

The comparatively high number of LoF mutations corresponding to premature termination codons prompted us to investigate whether these might involve CGA > TGA (Arg > Ter) transitions, which are particularly frequent in the human genome and compatible with a model of methylation-mediated deamination of 5mC (Cooper et al. 2010). In our dataset, we found 119 premature stop codons of which 31 involved CGA > TGA transitions (Additional Table 1). These transitions are important in that they can emerge as multiple independent events during evolution owing to the hypermutability of CpG dinucleotides, but have also been reported in a large number of human genes in association with heritable disease e.g. *F8* (Youssoufian et al. 1986) and *PAH* (Abadie et al. 1989). Thus, the possible recurrence of these mutations challenges our ability to ascertain whether they are maintained in the human genome as a consequence of identity by descent or are instead a product of the hypermutability of the CpG dinucleotides (identical by state).

### Polymorphic pseudogenes and the frequencies of LoF alleles in human populations

To assess the extent of gene loss, we performed cross-population analysis and ascertained the allele frequencies of the most frequent LoF alleles for each polymorphic pseudogene from the 1KGP database (Fairley et al. 2019) (Additional Table 2). In our analysis we identified cases in which the average global frequency of the LoF was below 1%. However, global average frequencies can be misleading because the allele in question may be completely absent from some populations yet well represented in other populations (LoF allele frequency > 1%). This was the case for six genes (*OR4A8*,* OR2AG1*,* CNTNAP3B*,* OR2S2*,* ADAM2* and *CFHR1*). Thus, we opted to include these genes in our analysis as polymorphic pseudogenes. One example is the *CFHR1* gene, which despite presenting a global frequency of the LoF variant (rs140799744) below 1%, the LoF allele was found exclusively in the African population where it attains a frequency of 3%. (Additional Table 2).

In addition, we found 3 genes (*OR4A8*,* OR2S2* and *CFHR1*) in which the LoF allele only appeared in one specific population (East Asian, South Asian and African respectively) (Fig. 1E in bold). Furthermore, in 10 genes (*SLC22A14*,* GUF1*,* OR4A8*,* COL6A5*,* EXO5*,* ANKRD36*,* EGF*,* OR2AG1*,* SPATA45*,* OR2S2)* the LoF allele was not present in the African population, suggesting that these LoF alleles originated more recently, after the dispersal of humans out of Africa (Fig. 1E underlined).

When considering the 179 polymorphic pseudogenes for which the LoF allele was found in all populations, in most cases the LoF allele was found to be fairly evenly distributed between the populations (Fig. 1F, Additional Fig. 2C) apart from *TRBV16* and *IFNL4*. In these latter two cases, the LoF allele attained a frequency of over 70% for *IFNL4* and 50% for *TRBV16* in the African population whereas in the remaining populations, the frequencies were considerably lower (< 40% for *IFNL4* and < 7% for *TRBV16*). The *IFNL4* gene encodes the interferon lambda 4 protein which has both a pro-viral and an anti-inflammatory role; the TT genotype identified in this work (rs11322783, also known as rs368234815 and ss469415590) may have been favoured through positive selection (Prokunina-Olsson et al. 2013). Individuals carrying the *IFNL4*-TT genotype do not produce the IFN-λ4 protein, and hepatitis C patients lacking IFN-λ4 exhibit higher rates of viral clearance. Further, the ability to produce IFN-λ4 appears to compromise immune protection against *Plasmodium falciparum* malaria in Kenyan children (Samayoa-Reyes et al. 2021).

**Fig. 2:**
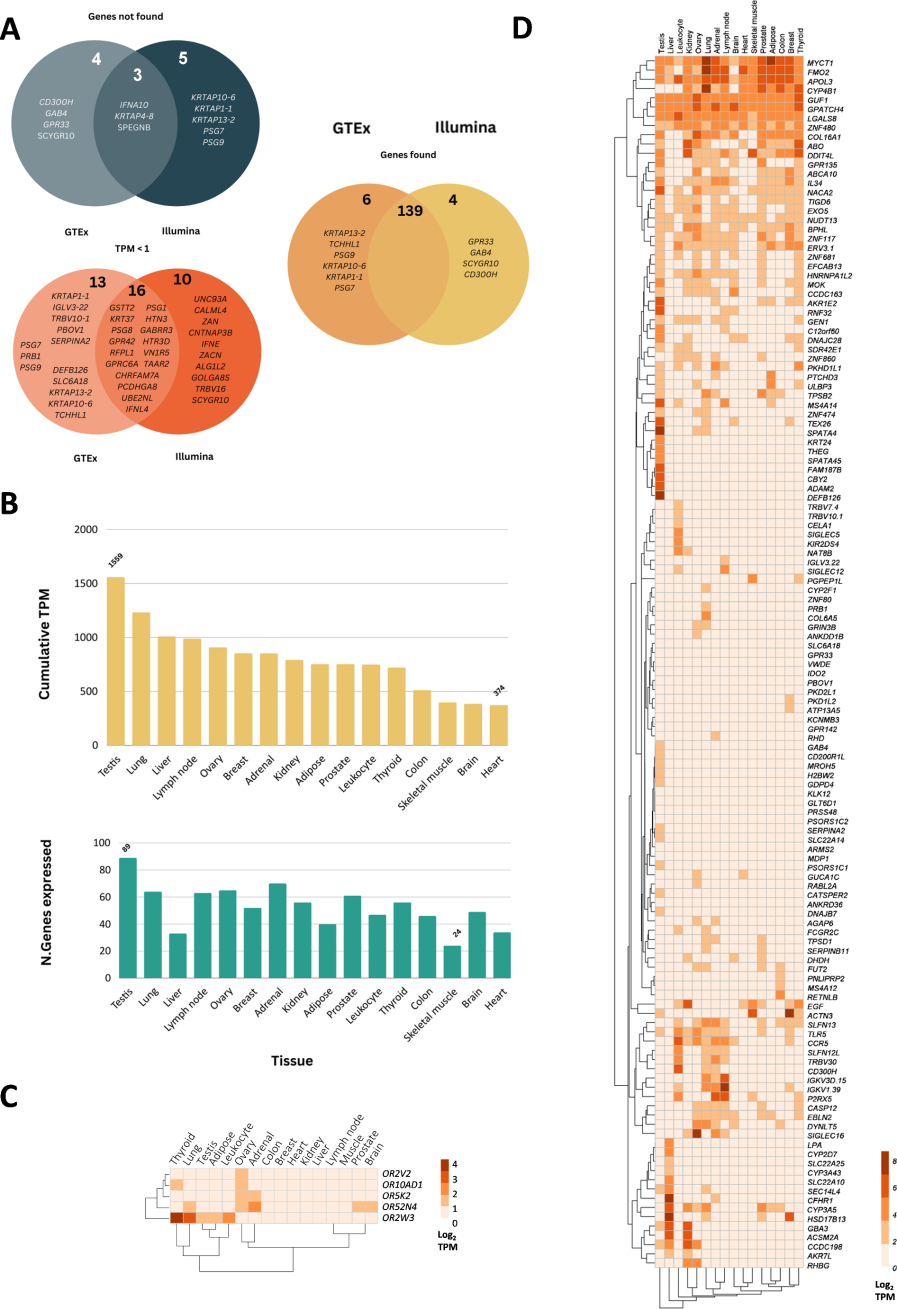
Illumina transcriptome analysis of human polymorphic pseudogenes. **A** - Comparative analysis of the Illumina transcriptome and GTEx transcriptome data. **B** - On top - cumulative TPM values per tissue in the Illumina transcriptome, on the bottom - number of pseudogenes expressed in each tissue **C** - Hierarchical clustering of human OR polymorphic pseudogenes in the Illumina transcriptome. **D** - Hierarchical clustering of human non-OR polymorphic pseudogenes in the Illumina transcriptome

This disparity of allele frequencies between populations is typically interpreted as a signal of selection or random genetic drift. The question of whether these two LoF alleles are favoured by selection in the African population over the coding allele, deserves further attention, although this goes beyond the scope of this study.

Notably, we found six genes containing LoF alleles whose global average frequencies exceeded 85% (*OR5AL1*,* OR10J4*,* OR8J2*,* CASP12*,* AKR7L* and *GPR33*). Such a high prevalence of non-functional gene copies in human populations indicates that the LoF alleles are nearing fixation and that eventually, these loci are likely to become non-coding. With respect to the OR genes, *OR5AL1*,* OR10J4* and *OR8J2* have been previously annotated as pseudogenes in the olfactory receptor database (Crasto et al. 2002).

Among the non-OR polymorphic pseudogenes are *GPR33* and *CASP12*, two genes with high frequencies of LoF alleles (97% for *GPR33* and 94% for *CASP12*) (Römpler et al. 2005; Dadley-Moore 2004). Interestingly, it has been argued that the inactivation of *CASP12* in humans might be protective against infection and sepsis, with the increase in LoF allele frequency being driven by positive selection (Dadley-Moore 2004). It has also been speculated that the inactivation of *GPR33* may have been adaptive, having been driven by an enhanced immune response to an unidentified pathogen (Bohnekamp et al. 2010). In addition to the proposed protective traits contributing to the high frequency of inactivation of these genes, we must also consider the possibility of recurrent mutation of CpG dinucleotides given that in both cases, the identified LoF mutation in the polymorphic pseudogene was a CGA > TGA (Arg > Ter) transition.

Of the 35 newly identified polymorphic pseudogenes, seven belonged to the OR multigene family whereas the remaining 28 were non-OR genes. From the set of newly identified polymorphic pseudogenes, the one with the highest LoF allele frequency was *PSG7* (LoF allele frequency = 86%) which exhibits exclusive expression in placental trophoblasts (Camolotto et al. 2010). The identified mutation in *PSG7* (p.Arg98Ter) corresponds to a premature stop codon that impacts all alternative transcripts. Currently, the biological function of the pregnancy-specific beta-1-glycoprotein 7 encoded by *PSG7* is unclear. Although differences in levels of PSG7, PSG9 and PSG1 proteins (all encoded by polymorphic pseudogenes) have been proposed as biomarkers for preeclampsia (Kandel et al. 2022; Toprak et al. 2023), no information regarding the individual genetic variability of these genes was provided in these studies. Of further note is PSG8, another member of this pregnancy-specific glycoprotein gene family and an additional polymorphic pseudogene. Thus, intriguingly four out of 11 *PSG* genes identified in the human genome are polymorphic pseudogenes.

**Table 1:** Newly identified polymorphic pseudogenes. (* Global average LoF allele frequency below 1% (0.01); however, the minimum frequency is attained in South Asian population, and one homozygote was identified)

Gene	LoF allele	rs code	Frequency of LoF allele	Frequency of homozygotes	RNA evidence	Protein evidence
*PSG7*	p.Arg98Ter	rs113247044	0.860		-	Deep proteome
*OR1P1*	p.Ter185Lys	rs7222006	0.718	0.52	-	-
*OR4K3*	p.Trp208LeufsTer109	rs5807006	0.614	0.335	-	-
*OR52P1*	p.Cys13ValfsTer174	rs34463490	0.608	0.376	-	-
*OR4C45*	p.Ter60Try	rs397826261	0.5584		-	-
*OR8G1*	p.Tyr259Ter	rs4268525	0.555	0.326	-	-
*OR4L1*	p.Ile83ThrfsTer10	rs33965693	0.532	0.306	-	-
*ANKDD1B*	p.Trp218Ter	rs34358	0.507	0.269	GTEx/Illumina	
*SIGLEC16*	p.Ter344Ser	rs8111526	0.505	0.285	GTEx/Illumina	-
*ALG1L2*	p.Leu157TrpfsTer5	rs55800015	0.453	0.181	GTEx	-
*GPATCH4*	p.Trp399Ter	rs3795733	0.436	0.229	GTEx/Illumina	Human proteome adult
*TPSB2*	p.Cys59Ter	rs72633259	0.399	0.204	GTEx/Illumina	-
*H2BW2*	p.Gln67Ter	rs2301384	0.306	XX- 0.064 XY- 0.148	GTEx/Illumina	-
*GSTT2*	p.Arg196Ter	rs201176441	0.272	0.224	-	Human proteome adult
*RHBG*	p.Trp100Ter	rs2245623	0.271	0.091	GTEx/Illumina	Human protein atlas
*IGLV3-22*	p.Glu63AspfsTer10	rs77364963	0.249		Illumina	-
*TRBV10-1*	p.Glu98Ter	rs17249	0.249	0.077	Illumina	-
*IDO2*	p.Tyr346Ter	rs4503083	0.23	0.063	Illumina	-
*VN1R5*	p.Ter46Gln	rs1778540	0.218	0.069	GTEx	-
*PRSS48*	p.Ser44ArgfsTer21	rs77216366	0.212	0.066	GTEx/Illumina	-
*ZAN*	p.Trp1883Ter	rs2293766	0.178	0.072	GTEx	-
*VWDE*	p.Arg385Ter	rs17165936	0.167	0.033	GTEx/Illumina	-
*TRBV30*	p.Arg54Ter	rs17267	0.144	0.029	GTEx/Illumina	-
*TRBV16*	p.Tyr50Ter	rs17284	0.1408		GTEx	-
*CYP4B1*	p.Asp295GlyfsTer3	rs3215983	0.135	0.03	GTEx/Illumina	Deep proteome
*SCYGR10*	p.Cys29Ter	rs144457324	0.113	0.018	-	-
*ZNF681*	p.Cys203TrpfsTer5	rs61397759	0.113	0.017	GTEx/Illumina	-
*IGKV1-39*	p.Arg18Ter	rs13392194	0.1068		GTEx/Illumina	-
*TAAR2*	p.Trp168Ter	rs8192646	0.059	0.009	GTEx	-
*SPEGNB*	p.Val64GlyfsTer26	rs11437065	0.058	0.005	-	-
*IGKV3D-15*	p.Trp114Ter	rs71241757	0.023	0.003	GTEx/Illumina	Deep proteome
*OR3A1*	p.Lys92Ter	rs7218125	0.022	0.001	-	-
*TRBV7-4*	c.50-1G > A (SA)	rs149835058	0.017	0.0003	GTEx/Illumina	-
*ATP13A5*	p.Gln355Ter	rs74437357	0.01	0.001	GTEx/Illumina	-
*ADAM2*	c.571–2 A > T (SA)	rs75268423	0.006*	0.0003	GTEx/Illumina	Human protein atlas

### Transcriptome and proteome analysis

Unless regulatory elements are compromised by inactivating mutations, polymorphic pseudogenes in principal retain the potential to be transcribed into RNA (even when harbouring the LoF variant). To demonstrate this, we collected and analysed data from human baseline transcriptomes (Illumina Body Map (Institute 2011) and GTEx (Consortium 2015) and proteomes (Human Protein Atlas (Kim et al. 2014; Uhlén et al. 2015), Human Proteome (Kim et al. 2014; Pinto et al. 2014) and Deep Proteome atlas (Wang et al. 2019). This process was performed separately for the non-OR and OR genes. Regarding the non-OR genes, we were unable to detect transcripts from 12 genes: 3 genes (*IFNA10*,* KRTAP4-8* and *SPEGNB*) were not found in both transcriptomes, 4 genes (*CD300H*,* GAB4*,* GPR33* and *SCYGR10*) were not detected in the GTEx dataset and 5 genes (*KRTAP10-6*,* KRTAP1-1*,* KRTAP13-2*,* PSG7* and *PSG9*) were not found in the Illumina body transcript dataset (Fig. 2A). A total of 39 genes revealed a TPM (transcripts per million) value below 1, 16 of which were shared by both transcriptome datasets. The absence or low level of transcription (TPM < 1) detected for these genes in the analysed transcriptomes may have been due to: (i) the high frequency of LoF mutations as exemplified by the *GPR33* (LoF allele frequency = ~ 97%), *PSG7* (LoF allele frequency = ~ 85%) and *UBE2NL* (LoF allele frequency = ~ 61%) gene loci, (ii) conditional gene expression; for example, the *IFNA10*,* IFNL4* and *IFNE* genes encoding various interferons are only expressed when challenged by viruses and other pathogens (iii) the expression profile is restricted in terms of developmental stage or tissue, for example, *PSG7* and *PSG1* are only expressed during pregnancy in placental trophoblasts or (iv) technical factors in the quantification of transcripts, given that Rozowsky et al., showed that mapping RNA-seq reads to personal genomes leads to a significant increase in the detection of RNA expression, opposed to mapping reads to reference genomes as performed in these projects (Rozowsky et al. 2023).

For the remaining non-OR genes, transcripts were detected in both transcriptomes. Analysis of the expression of these genes revealed that the tissue with the highest number of expressed polymorphic pseudogenes and also the highest number of TPM detected, was the testis (Fig. 2B). This finding is in agreement with previous works that also reported high level of expression of pseudogenes in testis (Rozowsky et al. 2023). In the Illumina transcriptome, testis showed a cumulative TPM of 1559 (the sum of all the TPM values of all genes expressed in each tissue), followed by lung, liver and lymph node. In the GTEx transcriptome, testis presented a cumulative TPM of 1703, although pancreas exhibited a higher cumulative TPM value (4997); this latter value was largely attributable to the tissue-specific expression level of the *PNLIPRP2* gene (Additional Fig. 3A and B). Of the five genes with the highest expression, three were coincident in both transcriptomes: *CYP4B1*,* FMO2* and *APOL3*. Conversely, no concordance between transcriptomes was found for the genes with the lowest expression, possibly due to different sensitivities and limits of detection between experiments. Heatmap analysis of expression per tissue in both datasets showed a similar hierarchical clustering for both transcriptomes (Fig. 2C and D and Additional Fig. 4A and B).

The 66 polymorphic OR pseudogenes revealed an overall low gene expression level by comparison with the non-OR dataset: only five genes were detected (TPM > 1) in the Illumina Body Map (*OR2V2*,* OR5K2*,* OR10A1*,* OR2W3* and *OR52N4*) and 6 in GTEx (*OR5G3*,* OR52D1*,* OR5K2*,* OR10AD1*,* OR2W3* and *OR52N4*) (Fig. 2C and Additional Fig. 4B). It must be stressed, however, that both transcriptome projects failed to include olfactory epithelium, a tissue which is known to be densely populated with olfactory receptors (Verbeurgt et al. 2014; Olender et al. 2016). The expression of ORs in non-olfactory tissues has also been reported in humans and other mammals (Jundi et al. 2023). Many of the G-protein‐coupled receptors (GPCR) encoded by OR genes have been demonstrated to exhibit ectopic expression in tissues such as the thyroid, testis, sperm and retina (Weidinger et al. 2021; Flegel et al. 2016; Milardi et al. 2017; Jovancevic et al. 2017). Although the function of extra-nasally expressed ORs remains to be clarified, evidence is accumulating that they are involved in a variety of different cellular processes (Weidinger et al. 2021). Thus, for example, in the testis the presence of OR in sperm was linked to chemotaxis, chemokinesis and recognition of other stimuli from the oocyte and surrounding cells (Flegel et al. 2016).

In this study, we found that in both transcriptomes the OR with the highest expression levels was *OR2W3*, as measured in thyroid, lung and testis (Illumina Body map) and blood and thyroid (in the GTEx), followed by *OR52N4* (Fig. 2C, Additional Fig. 4B).

Next, protein evidence (full and partial length peptides) was collected from three baseline human proteomes (Kim et al. 2014; Uhlén et al. 2015; Pinto et al. 2014; Wang et al. 2019). No evidence of translated protein was found for OR-encoding genes, but once again, none of the databases contained data from olfactory epithelium and/or olfactory bulb tissue, and so the observations were subject to a critical bias in terms of the tissues available to examine. Regarding the non-OR polymorphic pseudogenes, evidence of translated protein was found for 16 genes in the Human Protein Atlas (Kim et al. 2014; Uhlén et al. 2015), 21 in the Human Proteome Map Adult and Fetus (Kim et al. 2014; Pinto et al. 2014) and 39 in the Deep proteome atlas (Wang et al. 2019) (Additional Table 3). Combining the 3 proteome datasets, we found evidence for mRNA translation into protein for 54 genes. The tissues in which proteins emanating from polymorphic pseudogenes were detected most frequently were the testis, liver and pancreas, suggesting that these specific tissues may have a higher rate of expression or that the encoded proteins may play a more prominent role in the function of these tissues.

### Metabolic pathways affected by polymorphic pseudogene loss

To obtain insight into the main metabolic pathways that might be affected by polymorphic pseudogene loss, we performed an enrichment analysis in three libraries (Wikipathway, Reactome, KEGG), which again was performed separately for the 166 non-OR and 66 OR polymorphic pseudogenes. The interrogated databases included annotations for 165 non-OR and 59 OR polymorphic pseudogenes, but not for *KRTAP28-10*,* OR13C7; OR1P1; OR4A8; OR52P1; OR5AC1; OR5G3; OR5H8*, making it impossible to consider these loci later in the analysis.

With respect to the non-OR genes, significant enrichment was found, with the top scoring pathway in all libraries analysed being pathways related to detoxification and xenobiotic metabolism (Table 2) involving the following genes: *CYP2D7*,* DHDH*,* CY3A43*,* GSTT2*,* AKR7L*,* CYP3A5*,* GSTT2*,* CYP2F1*,* ACSM2A*,* BPHL* and *CYP4B1*. Other pathways also found to be enriched were those related to various blood group systems (*FUT2*,* RHD*,* ABO*), keratinization (*KRTAP13-2*,* KRTAP4-8*,* KRTAP10-6*,* KRT37*,* KRT24*,* KRTAP1-1*, *KLKL12)* and the interferon-mediated pathway (*IFNE*,* IFNL4*,* IFNA10*). Other pathways worthy of note are those involved in phototransduction (*GUCA1C*,* CALML4*), salivary secretion (*HTN3*,* PRB1*,* CALML4*), neurotransmission (*GABRR3*,* GRIN3B*), fertilization, reproduction and sperm motility (*ADAM2*,* CATSPER2*,* SERPINA2*) (Additional Table 4).

Not surprisingly, enrichment analysis for the OR genes showed highly significant results for the pathways involved in the expression and translocation of olfactory receptors, olfactory transduction, olfactory signalling, and sensory perception (Table 2).

**Table 2:** Enrichment analysis p and q values rounded up to the nearest decimal point. Only *p*-values below 0.05 were considered: q ≤ 0.05** highly significant, q ≤ 0.1 or adjusted *p*-value ≤ 0.1 *significant. Unrounded values, Z-scores and combined scores are available in Additional Table 4

Metabolic pathways					
	Term	Library	*p*-value	q-value	Genes
Non-Olfactory receptor	Metabolism of xenobiotics by cytochrome P450	KEGG	0.00004**	0.005*	*CYP2D7*,* DHDH*,* AKR7L*,* CYP3A5*,* GSTT2*,* CYP2F1*
	Biological Oxidations R-HSA-211859	Reactome	0.0004*	0.07*	*ACSM2A*,* CYP2F1*,* CYP3A5*,* GSTT2*,* AKR7L*,* BPHL*,* CYP3A43*,* CYP4B1*
	Blood Group Systems Biosynthesis R-HSA-9033658	Reactome	0.0007*	0.07*	*FUT2*,* RHD*,* ABO*
	Xenobiotics R-HSA-211981	Reactome	0.0009*	0.07*	*CYP2F1*,* CYP3A5*,* CYP3A43*
	Phase I - Functionalization Of Compounds R-HSA-211945	Reactome	0.001*	0.07*	*CYP2F1*,* CYP3A5*,* BPHL*,* CYP3A43*,* CYP4B1*
	Keratinization R-HSA-6805567	Reactome	0.001*	0.07*	*KRTAP1-1*,* KRT37*,* KRT24*,* KRTAP4-8*,* KLK12*,* KRTAP13-2*,* KRTAP10-6*
	Oxidation by Cytochrome P450 WP43	WikiPathway	0.001*	0.1	*CYP3A5*,* CYP2F1*,* CYP4B1*,* CYP3A43*
	Overview of interferons-mediated signalling pathway WP4558	WikiPathway	0.003*	0.1	*IFNE*,* IFNL4*,* IFNA10*
	Metapathway biotransformation Phase I and II WP702	WikiPathway	0.004*	0.1	*CYP2F1*,* CYP3A5*,* GSTT2*,* CYP3A43*,* CYP4B1*,* FMO2*
Olfactory receptor	Expression And Translocation Of Olfactory Receptors R-HSA-9752946	Reactome	2.4e-89**	7.4658e-89	All except- *OR52B4*,* OR12D1*
	Olfactory transduction	KEGG	7.4e-89**	7.4707e-89	All except- *OR52B4*,* OR12D1*
	Olfactory Signalling Pathway R-HSA-381753	Reactome	8.5e-89**	1.2834e-88	All except- *OR8J2*
	Sensory Perception R-HSA-9709957	Reactome	1.5e-77**	1.491e-77	All except- *OR52B4*,* OR12D1*
	GPCRs, Class A Rhodopsin-like WP455	WikiPathway	0.00002**	0.00007	*OR2AG1*,* OR2S2*,* OR1D5*,* OR2F1*,* OR10J1*,* OR3A1*,* OR2D2*
	GPCRs, Other WP117	WikiPathway	0.003**	0.005	*OR3A1*,* OR8G1*,* OR2F1*

### Gene essentiality and genetic redundancy

To address the impact of gene essentiality, we searched the OGEEv3 gene essentiality database (Gurumayum et al. 2020). For each gene, we collected gene essentiality scores from three CRISPR gene knockout experiments performed in human cell lines (Additional Table 6). This revealed that when information was available, the genes sought were invariably deemed to be non-essential. The only possible exception was the *AGAP6* gene for which conflicting results were obtained, with two experiments (Avana and Sanger) ranking this gene as non-essential and one (GeCKO) classifying it as essential. Gene essentiality is a conditional trait dependent upon several factors such as the environmental and genomic context of the gene. Duplicated genes have previously been associated with genetic robustness against null mutations by providing genetic buffering through functional compensation and/or redundant metabolic pathways (El-Brolosy and Stainier 2017; Gu 2003; Gu et al. 2003). In addition, since duplicate genes that encode isoenzymes may mask haploinsufficiency or loss of function of duplicated gene copies, we next investigated if the polymorphic pseudogenes identified in this work presented gene duplicates/paralogues. This investigation (Additional Table 7) showed that 38 polymorphic pseudogenes were either single copy or represented ancient paralogues (preceding the origin of the chordate lineage over 500MYA – data not shown) for which sequence and functional redundancy is less likely. For the remaining 128 genes, at least one paralogue was found, which suggests that genetic redundancy may have played a key role in compensating for the inactivation of one paralogue; nonetheless, this should be validated on a case-by-case basis. In line with this thesis, we found 20 polymorphic pseudogenes with human-specific paralogues (i.e. duplicated in the human ancestral lineage after divergence from the chimpanzee lineage), most of which exhibited a relatively high frequency of inactivation, ranging from 96% for *AKR7L* to 11.2% in the case of *GOLGA8S*, possibly due to high sequence and functional redundancy. This analysis was not performed for the OR genes given that they belong to a highly duplicated gene family.

Finally, the OMIM database was searched to establish whether the identified polymorphic pseudogenes have been linked to specific Mendelian disorders. The search retrieved OMIM annotations for the following genes: *CASP12*,* TLR5*,* ACTN3*,* HSD17B13*,* CCR5*,* ARMS2*,* CFHR1*,* CYP3A5*,* GUF1*,* LPA* and *OR2W3* (Additional Table 5), and in the case of the *ACTN3*,* CASP12*,* CCR5*,* LPA* and *TLR5* genes, the LoF variant identified in OMIM was the same LoF identified in this work. Among the annotations found in OMIM, several were reportedly associated with beneficial outcomes. This was the case with the previously mentioned *CASP12* gene, which harbours LoF alleles associated with reduced risk of infection and sepsis (Fischer et al. 2002; Yeretssian et al. 2009), or *TLR5*, whose deficiency was associated with reduced organ failure and improved survival in melioidosis (West et al. 2013) as well as with resistance to systemic lupus erythematosus (Hawn et al. 2005). Another example is *ACTN3*, in which both disrupted and non-disrupted alleles of the gene can give rise to positive outcomes. In fact, the truncated *ACTN3* has been linked to increased cold endurance (Wyckelsma et al. 2021), whereas the non-disrupted *ACTN3* is reportedly associated with sprint performance (Yang et al. 2003). These cases demonstrate that the introduction of the LoF variant is not necessarily disadvantageous because selective pressure is critically dependent upon specific environmental conditions or stimuli. Similarly, the loss of the CCR5 receptor might result in both beneficial and detrimental outcomes, namely protection from infection by HIV (Samson et al. 1996) and hepatitis C virus (Goulding et al. 2005), protection against type 1 diabetes (Buhler et al. 2002) but an increased risk of developing symptomatic infection by West Nile virus (Glass et al. 2006). A LoF variant (rs72613567) in *HSD17B13* has been linked to a reduced risk of chronic liver and non-alcoholic fatty liver disease (Abul-Husn et al. 2018). The remaining annotations covered polymorphic pseudogenes either linked to a provisional phenotype (e.g. *GUF1*), or multifactorial diseases (e.g. *CYP3A5*), or with late onset diseases such as macular degeneration (e.g. *ARMS2*) (Fritsche et al. 2008), retinitis pigmentosa and azoospermia or oligospermia, OR2W3 (Ma et al. 2015; Aston et al. 2010).

## Discussion

Here we present a curated compendium of human polymorphic pseudogenes for which at least one homozygous LoF (natural knock-out) individual was identified in a healthy population sample (1KGP). This collection of polymorphic pseudogenes includes 66 olfactory receptor genes and 166 non-olfactory receptor genes, of which 35 are, to the best of our knowledge, reported here for the first time as polymorphic pseudogenes (Table 1). The remaining genes were either previously identified in studies that targeted individual genes (see Additional Table 1) or in analyses that identified multiple polymorphic pseudogenes such as Abascal et al. (Abascal et al. 2018), and Rausell et al. (Rausell et al. 2020). When comparing methodologies for the identification of polymorphic pseudogenes employed in previous works (Abascal et al. 2018; Rausell et al. 2020) with the approach described here, we find three different yet complementary strategies. Abascal et al., employed a comparative approach to identify genes with discrepancies in coding status by comparing gene sets from three databases (GENCODE v24, NCBI RefSeq 107, UniProtKB). Rausell et al., on the other hand, identified polymorphic pseudogenes by focusing on loss-of-function (LoF) variants and their allelic frequencies using population genomic data from the gnomAD database. In the present work, the strategy adopted was to identify polymorphic pseudogenes using the automatic annotations in NCBI and GeneCards^®^, which label genes with ambiguous coding status as “gene/pseudogene”. Although the identification strategies employed varied between the current work and previous studies, the combination of the results showed little overlap (12 genes, Fig. 1A) evidencing the advantage in combining different approaches to obtain a more comprehensive list of human polymorphic pseudogenes.

In this work, we additionally provide evidence for the expression of identified polymorphic pseudogenes at both mRNA and protein levels, a strong indication that these genes remain active and may play a functional role in those humans who harbour the intact coding allele. Interestingly, we found genes with both high LoF allele frequencies (above 40%) and high transcription levels such as: *ZNF117*,* GPATCH4* and *PNLIPR2*. This finding raises the question of whether such encoded RNA transcripts might play a regulatory role, for instance, of small RNA genes (Pink et al. 2011). Still, we were able to obtain evidence for translated protein products for *GPATCH4* and *PNLIPR2*, thereby proving that when harbouring the coding allele these genes produce an mRNA that is translated. Further, to gain insight into the main metabolic pathways affected by polymorphic gene loss, enrichment analysis was performed. Polymorphic pseudogenes were found to contribute to additional pathways such as xenobiotic/drug metabolism and detoxification pathways, blood type determination, keratinization and the immune response. A similar analysis has been previously reported using gene ontology libraries and a significant enrichment in olfactory receptor genes was detected (Rausell et al. 2020).

As a rule, the pharmacogenomics field is focused on the interaction between genetic polymorphisms and drugs, with the focus being mainly on copy number variants, transcription levels of genes involved in the drug uptake and/or metabolism, and missense mutations in genes that encode proteins involved in drug response. In this work, we provide evidence that polymorphic pseudogenes may also be a significant factor to be considered when evaluating an individual’s pharmacogenomic profile. We deduce that several genes from the Cytochrome oxidase P450 gene family are polymorphic pseudogenes, with their LoF alleles being well represented in the global population *CYP4B1* (13.5%), *CYP2D7* (38.9%), *CYP2F1* (27%), *CYP3A43* (10.5%) and *CYP3A5* (3%). Such a layer of variability in the genetic repertoire involved in the detoxification pathways may account for the intra-individual differences in the metabolism of drugs used in common therapeutic protocols in humans. A classic example is the *CYP2D6* gene which is characterized by copy number variation: the number of gene copies present in the genome is predictive of the metabolizing phenotype, ranging from poor to ultra-rapid metabolizers (Demkow 2016). Other examples of polymorphic pseudogenes involved in drug metabolism are *BPHL and FMO2. BPHL* encodes a hydrolase that activates the prodrug valacyclovir to acyclovir and hydrolyzes other drugs such as zidovudine, floxuridine and gemcitabine (Lai et al. 2008). *FMO2* catalyses the oxygenation of the antitubercular drugs, thioacetazone and ethionamide; a cross-population analysis has revealed a relatively high frequency of the *FMO2* LoF allele in the African population (~ 30%) as compared to other populations where it attains a maximum of ~ 5%. Interestingly, toxicity and/or lack of effect of thioacetazones have previously been reported in African populations (Elliott and Foster 1996; Francois et al. 2009). In the case of *BPHL*, the LoF allele has only been observed in three populations (African, American and East Asian) of which the African population has a comparatively high LoF frequency (~ 15%), a finding which may help to explain the lower efficacy of acyclovir in Africans (Lu et al. 2012). In addition to xenobiotic metabolism, we also found that polymorphic pseudogenes were significantly overrepresented in the pathways related to immunity, more specifically to interferon-mediated signalling, underscoring the intra-individual variability in the immune response observed within and between human populations (Gagneux 2012; Sabri et al. 2014).

### Study considerations and limitations

The methodology adopted to identify polymorphic pseudogenes in the present study represents a conservative approach that will have certainly underestimated the true number of polymorphic pseudogenes in the human genome. In our study, we did not consider missense mutations in the coding region, many of which could disrupt protein function, although this would be hard to confirm unambiguously. Nor did we consider mutations in the regulatory and non-coding regions that could abolish gene expression. Our approach also did not include large insertions or deletions that encompass whole exons and entire genes (copy number variants). Alterations to canonical splice sites were however considered to constitute deleterious LoF variants as they may be predicted to disrupt the transcription of the canonical/major isoform of the target gene. Given that only one LoF mutation (with the highest global frequency) per polymorphic pseudogene was taken into consideration, this may have led to an underestimation of the frequency of gene loss given that other LoF mutations co-occurring in the same gene were not considered.

## Conclusion

The identification and analysis of human polymorphic pseudogenes and their corresponding LoF variants across the global population and corresponding sub-populations, provides valuable insights into the universality and/or specificity of these genetic factors. The almost individual variability of the human genetic repertoire adds another level of complexity to the human genome that is crucial to consider in the context of human health. The genetic diversity introduced by polymorphic pseudogenes may contribute to the individual variable response to xenobiotic/drug metabolism and ongoing co-evolutionary dynamics between the host and pathogens. Interestingly, many polymorphic pseudogenes are members of multigene families, and this suggests that genetic redundancy is likely to play a key role in compensating for the inactivation of a given paralogue. In addition, the introduction of the LoF variant is not necessarily disadvantageous because selective advantage/disadvantage will be critically dependent upon the environmental context and stimuli encountered by the individual concerned.

This work presents an updated manually curated dataset of human polymorphic pseudogenes. Nevertheless, we predict that the number of identified polymorphic pseudogenes will increase as increased efforts are directed toward genome sequencing and the characterization of the lexicon of coding sequences.

## Methods

### Collection and identification of human candidate polymorphic pseudogenes

Polymorphic pseudogenes were collated from the literature including those listed by Abascal et al. (Abascal et al. 2018) and by Rausell et al. (Rausell et al. 2020). To identify novel unreported and hitherto unrecognized polymorphic pseudogenes, the NCBI gene database (Brown et al. 2014) was searched using “gene/pseudogene” as a query; the results were filtered so as to contain only sequences from *Homo sapiens* from the current RefSeq collection. GeneCards^®^, the human gene database (Safran et al. 2021; Stelzer et al. 2016), was also searched using the same keyword combination. The results from these searches were combined into a single non-redundant list of putatively polymorphic human pseudogenes (Additional Table 1).

### Validation of putative polymorphic pseudogenes using 1000 genome project (1KGP) data

Each candidate polymorphic pseudogene was inspected individually to identify the LoF mutation it contained and to determine the corresponding frequency of this variant in the human population. The candidate genes were validated as *bona fide* polymorphic pseudogenes if (i) they harboured at least one LoF mutation (frameshift, premature stop codon, or disruption of one of the canonical splice sites) in the coding region, (ii) they harboured the LoF mutation in the canonical/predominant transcript, (iii) they displayed homozygosity for the LoF mutation in at least one individual, and (iv) the LoF mutation attained a frequency ≥ 1% (0.01) in at least one population (See Additional Fig. 1). To obtain these data from a healthy cohort, we screened the 1000 Genomes Project Phase 3 (1KGP) (Auton et al. 2015) using the Ensembl genome browser (release 110 July 2023) variant table. The 1KGP was selected as it contains allele and genotype frequencies of 4973 healthy adults (over 18 years) from 26 populations (Fairley et al. 2019). Gene variant data tables were filtered to show (i) data from the 1KGP and (ii) LoF variants (i.e. excluding variants in non-coding regions and missense variants) and (iii) variants contained within the canonical/predominant transcript (i.e. excluding variants localized in alternatively spliced exons). For each gene, the LoF mutation with the highest allele frequency was identified, ignoring additional LoF variants with lower frequencies. When 1KGP frequency data were not available from the Ensembl pipeline, we collected the 1KGP data available in the Genome Aggregation Database-gnomAD v4.0.0 (Karczewski et al. 2020).

### Analysis of LoF allele frequency and population heterogeneity

Frequencies of the identified LoF alleles were collated from the 1KGP (Auton et al. 2015) for the following populations: African (African Caribbean in Barbados, African ancestry in the Southwestern US, Esan in Nigeria, Gambian in Western Division, Luhya in Webuye, Mende in Sierra Leone, Yoruba in Ibadan); American (Colombian in Medellin, Mexican ancestry in Los Angeles, Peruvian in Lima, Puerto Rican in Puerto Rico); East Asian (Chinese Dai in Xishuangbabba, Han Chinese in Beijing, Southern Han Chinese, Japanese in Tokyo, Kinh in Ho Chi Minh City); European (Utah residents with Northern and western European ancestry, Finnish in Finland, British in England and Scotland, Iberian populations in Spain, Tuscany in Italy) and South Asian (Bengali in Bangladesh, Gujarati Indian in Houston, Indian Telugu in the UK, Punjabi in Lahore, Sri Lankan Tamil in the UK) (Additional Table 2).

### Transcriptome, proteome and enrichment analysis

Baseline healthy human transcriptome data were collected from the GTEx consortium (Consortium 2015) and Illumina Body Map Project (Institute 2011). Baseline healthy human proteome data were collected from the Human Protein Atlas project (Kim et al. 2014; Uhlén et al. 2015), Human Proteome map (Kim et al. 2014; Pinto et al. 2014) and from Wang et al., Deep Proteome Atlas (Wang et al. 2019). All data were collected via the EMBL-EBI Expression atlas (Moreno et al. 2021) using the Human Ensembl gene IDs. Gene expression data were at cutoff TPM > 1 so as to exclude low identity and immature RNA reads, whilst proteome data were cut off at 1ppb (part per billion). Hierarchically clustered diagrams of gene expression were calculated with Log2 transformed TPM values, using pheatmap clustering in RStudio build 494 (R version 4.3.1). Enrichment analysis was run across three metabolic libraries: Reactome release 2022 (Gillespie et al. 2021), Wikipathways Human database (Agrawal et al. 2023), KEGG pathways human database 2021 release (Kanehisa and Goto 2000), using Enrichr-KG (Evangelista et al. 2023). Significant results were considered when *p*-values were ≤ 0.05 and q-values were ≤ 0.1. A less stringent q-value or adjusted *p*-value cutoff was set to allow a broader exploration of potential associations and relationships.

### Gene essentiality and paralogue analysis

Gene essentiality was investigated using the database OGEEV3 available online (Gurumayum et al. 2020); genes were searched individually, and gene essentiality scores were collected for CRISPR experiments (Additional Table 6). Paralogues were collected for each non-OR gene using the PantherDB Tools Ortholog/homologOther via the Panther services open API, Panther DB version 18.0 (Thomas et al. 2022). Paralogues were filtered so as to include the paralogues ranging from Euteleostomi to Human-specific.

## Electronic Supplementary Material

Below is the link to the electronic supplementary material.

Supplementary Material 1

Supplementary Material 2

Supplementary Material 3

Supplementary Material 4

Supplementary Material 5

Supplementary Material 6

Supplementary Material 7

Supplementary Material 8

## Data Availability

Data is provided within the manuscript or supplementary information files.

## Abbreviations

OR: Olfactory receptor
