# Supplementary material for: Polymorphic pseudogenes in the human genome - a comprehensive assessment
Source: Hum Genet. 2024 Nov 2;143(12):1465–79. doi: 10.1007/s00439-024-02715-9 (PMC11576641; doi:10.1007/s00439-024-02715-9)
Supplement: Supplementary file 5 — Supplementary Material 5 [file 439_2024_2715_MOESM5_ESM.docx]

**Additional Table 5:** Results of the OMIM database search for geno-phenotype annotations for the identified polymorphic pseudogenes. * Provisional association.

| Gene | Phenotype MIM number | Phenotype |
| --- | --- | --- |
| *ACTN3* | 617749 | Alpha-actinin-3 deficiency  Sprinting performance  Increased cold tolerance |
| *ARMS2* | 613778 | Macular degeneration, age-related, 8 |
| *CASP12* | - | Sepsis, susceptibility to |
| *CCR5* | 612522  609532  609423  610379 | Diabetes mellitus, insulin-dependent, 22  Hepatitis C virus, resistance to  HIV infection, susceptibility/resistance to  West Nile virus, susceptibility to |
| *CFHR1* | 235400  603075 | Hemolytic uremic syndrome, atypical, susceptibility to  Macular degeneration, age-related, reduced risk of |
| *CYP3A5* | 145500 | Hypertension, salt-sensitive essential, susceptibility to |
| *GUF1* | 617065 | Developmental and epileptic encephalopathy 40 * |
| *HSD17B13* | 620116 | Fatty liver disease, protection from |
| *LPA* | 618807 | LPA deficiency, congenital  Coronary artery disease, susceptibility to |
| *TLR5* | 608556  615557  601744 | Legionnaire disease, susceptibility to  Melioidosis, susceptibility to  Systemic lupus erythematosus, resistance to  Systemic lupus erythematosus, susceptibility to, 1 |
| *OR2W3* | 268000  616729 | Possible association with retinis pigmentosa *  Risk for Azoospermia and oligozoospermia |
