# Supplementary material for: Polymorphic pseudogenes in the human genome - a comprehensive assessment
Source: Hum Genet. 2024 Nov 2;143(12):1465–79. doi: 10.1007/s00439-024-02715-9 (PMC11576641; doi:10.1007/s00439-024-02715-9)
Supplement: Supplementary file 8 — Supplementary Material 8 [file 439_2024_2715_MOESM8_ESM.pptx]

## Slide 1
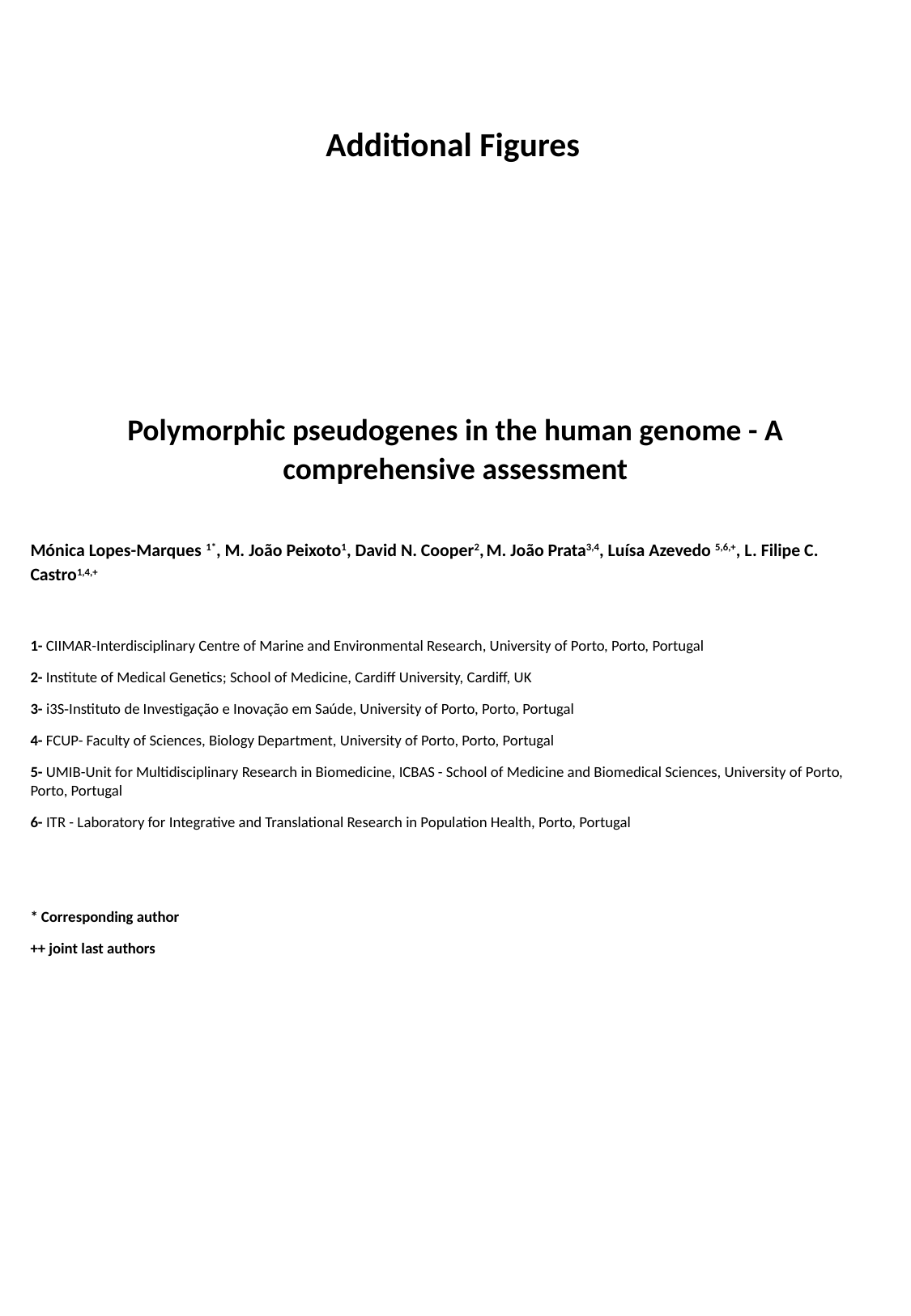

Additional Figures
Polymorphic pseudogenes in the human genome - A comprehensive assessment
Mónica Lopes-Marques 1*, M. João Peixoto1, David N. Cooper2, M. João Prata3,4, Luísa Azevedo 5,6,+, L. Filipe C. Castro1,4,+
1- CIIMAR-Interdisciplinary Centre of Marine and Environmental Research, University of Porto, Porto, Portugal
2- Institute of Medical Genetics; School of Medicine, Cardiff University, Cardiff, UK
3- i3S‐Instituto de Investigação e Inovação em Saúde, University of Porto, Porto, Portugal
4- FCUP- Faculty of Sciences, Biology Department, University of Porto, Porto, Portugal
5- UMIB-Unit for Multidisciplinary Research in Biomedicine, ICBAS - School of Medicine and Biomedical Sciences, University of Porto, Porto, Portugal
6- ITR - Laboratory for Integrative and Translational Research in Population Health, Porto, Portugal
* Corresponding author
++ joint last authors

## Slide 2
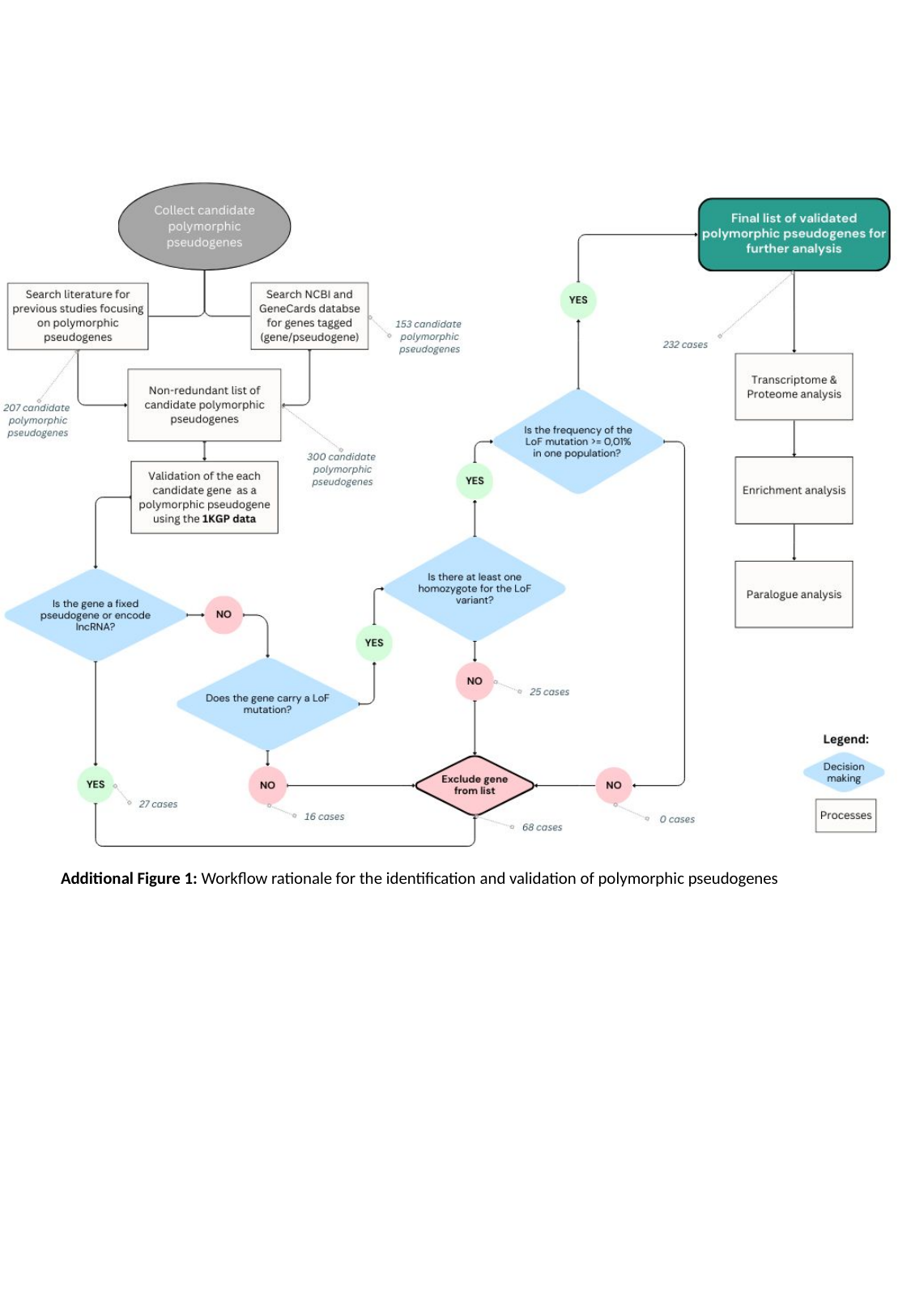

Additional Figure 1: Workflow rationale for the identification and validation of polymorphic pseudogenes

## Slide 3
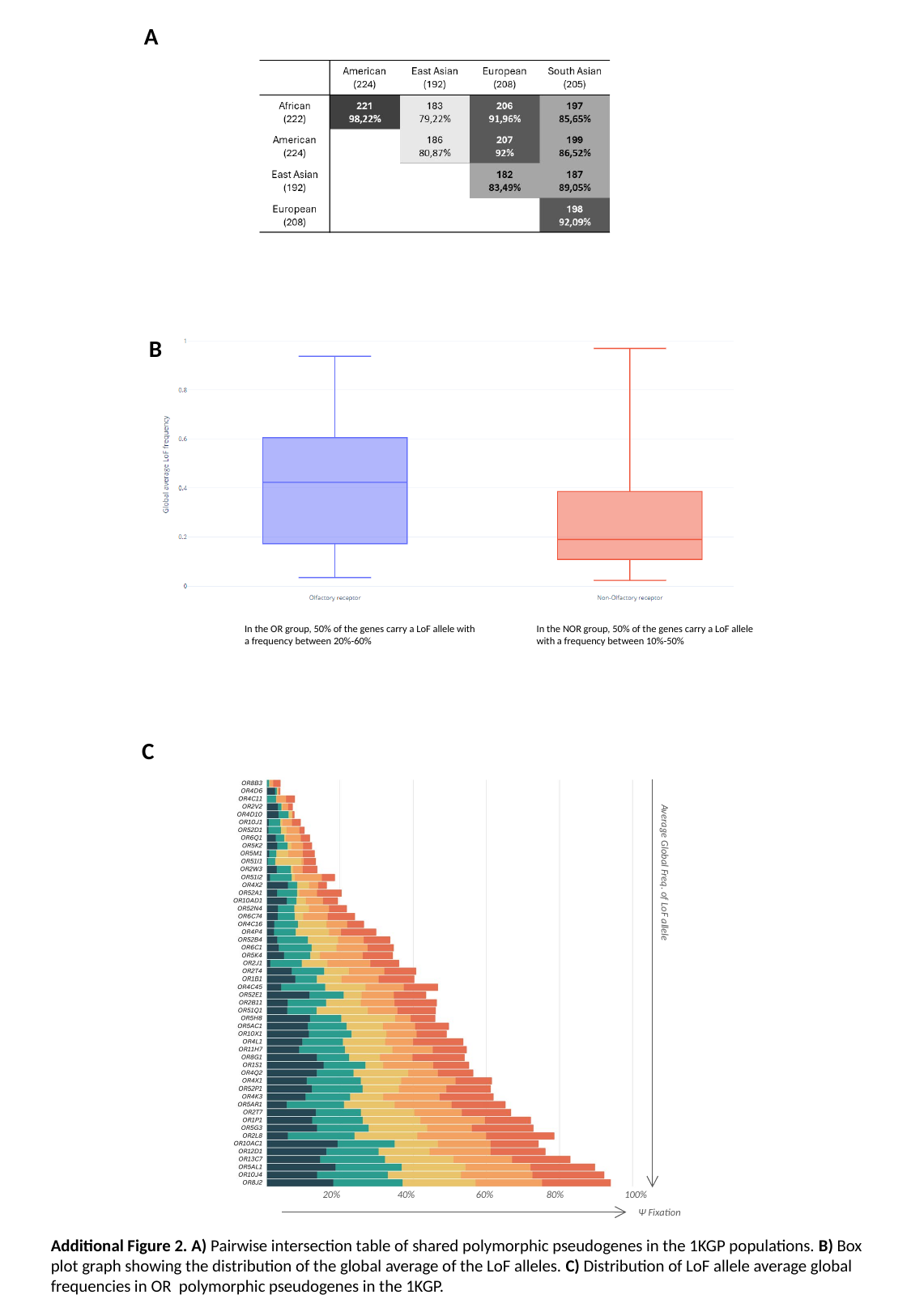

A
B
In the OR group, 50% of the genes carry a LoF allele with a frequency between 20%-60%
In the NOR group, 50% of the genes carry a LoF allele with a frequency between 10%-50%
C
Average Global Freq. of LoF allele
20%
40%
60%
80%
100%
Ψ Fixation
Additional Figure 2. A) Pairwise intersection table of shared polymorphic pseudogenes in the 1KGP populations. B) Box plot graph showing the distribution of the global average of the LoF alleles. C) Distribution of LoF allele average global frequencies in OR polymorphic pseudogenes in the 1KGP.

## Slide 4
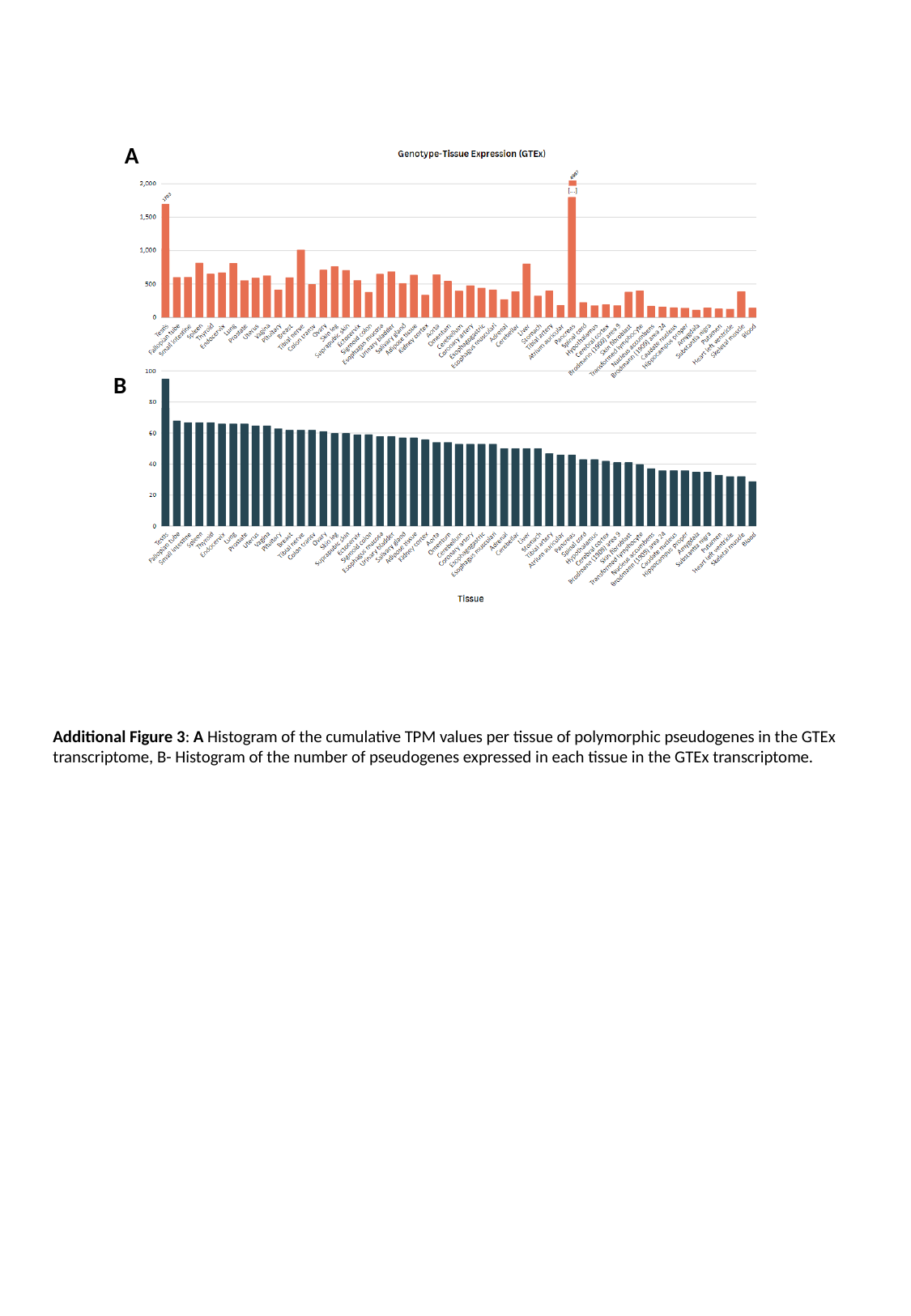

A
B
Additional Figure 3: A Histogram of the cumulative TPM values per tissue of polymorphic pseudogenes in the GTEx transcriptome, B- Histogram of the number of pseudogenes expressed in each tissue in the GTEx transcriptome.

## Slide 5
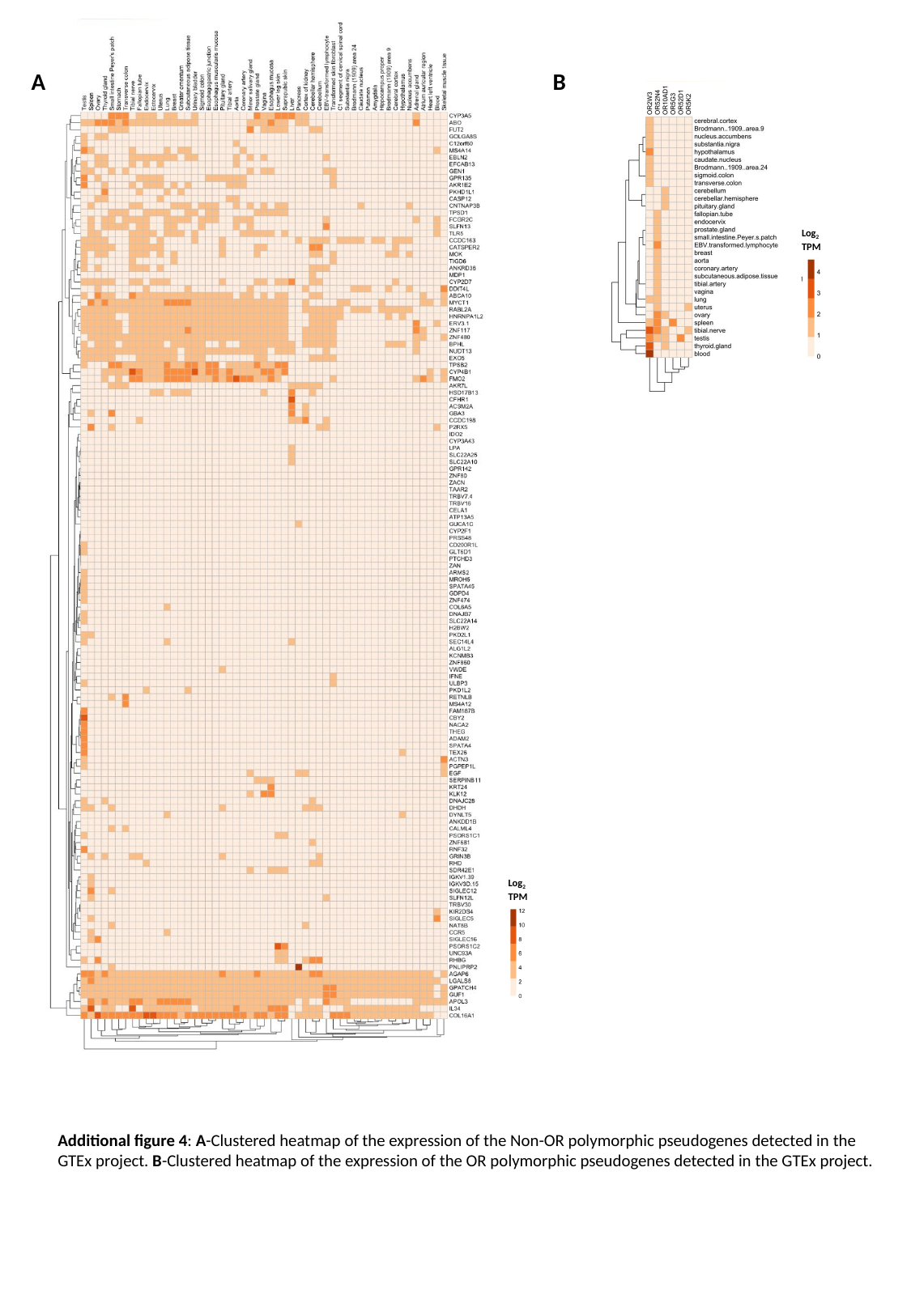

A
B
Log2 TPM
Log2 TPM
Additional figure 4: A-Clustered heatmap of the expression of the Non-OR polymorphic pseudogenes detected in the GTEx project. B-Clustered heatmap of the expression of the OR polymorphic pseudogenes detected in the GTEx project.
